# Ticks and Associated Rickettsiae from Domestic Animals in Bhutan

**DOI:** 10.3390/pathogens14101021

**Published:** 2025-10-08

**Authors:** Tshokey Tshokey, Mythili Tadepalli, Stephen R. Graves, John Stenos

**Affiliations:** 1 Australian Rickettsial Reference Laboratory (ARRL), University Hospital, Geelong, VIC 3220, Australia; mythili.tadepalli@barwonhealth.org.au (M.T.); graves.rickettsia@gmail.com (S.R.G.); john.stenos@barwonhealth.org.au (J.S.); 2 Department of Laboratory Medicine, Jigme Dorji Wangchuck National Referral Hospital (JDWNRH), Thimphu 11001, Bhutan; 3 College of Medicine and Public Health, Flinders University, Mount Gambier, SA 5290, Australia

**Keywords:** Bhutan, domestic animals, phylogenetic analysis, rickettsiae, ticks

## Abstract

In Bhutan, information on rickettsiae is limited to a few epidemiological studies. There is no information on ticks and tick-associated rickettsiae. Ticks were collected opportunistically from domestic animals residing in eight districts where a seroprevalence study had been carried out previously. Morphological identification of the ticks was performed in the United States National Tick Collection and testing for rickettsiae was carried out in the Australian Rickettsial Reference Laboratory. Samples positive for rickettsiae by qPCR were subjected to conventional PCR followed by DNA sequencing and phylogenetic analysis. A total of 200 ticks were sampled from 155 domestic animals including cattle, dogs, goats, horses, yaks, sheep and cats. The ticks belonged to twelve different species, the commonest being *Rhipicephalus microplus*, followed by *Rhipicephalus haemaphysaloides*, *Haemaphysalis* sp. near *ramachandrai*, *Haemaphysalis tibetensis*, *Haemaphysalis bispinosa*, *Haemaphysalis* sp., *Haemaphysalis* sp. near *davisi*, *Rhipicephalus sanguineus*, *Haemaphysalis shimoga*, *Haemaphysalis hystricis*, *Ixodes ovatus*, and *Amblyomma testudinarium*. Rickettsial DNA sequence analysis showed that the rickettsiae infesting ticks in Bhutanese domestic animals aligned with *R. gravesii*, *R. canadensis*, *R. honei*, *R. africae*, *R. felis*, *R. akari*, *R. australis*, *R. japonica*, *R. africae*, *R. heilongjiangensis*, *R. conorii*, *R. peacockii*, *R. honei*, *R. massiliae* and *R. rhipicephali*. However, these may not be conclusive due to low bootstrap values in the phylogenetic tree. Bhutan will benefit from larger studies on ticks and tick-borne infections, burden and damage assessment to livestock and human health, public health interventions and clinical guidelines to reduce morbidity and mortality in human and animal health.

## 1. Introduction

Ticks transmit a larger variety of pathogenic microorganisms including protozoa, bacteria (including rickettsiae and spirochaetes) and viruses compared to all other arthropod vectors (except mosquitos) making them among the most important vectors of diseases affecting livestock, domestic animals and humans [1]. Humans become accidentally infected with these organisms through bites of infected ticks [2]. The consensus list of ticks resulting from recent studies have listed 896 tick species and three families although widespread disagreements exist [3] and new proposed tick species continue to be put forward [4]. India alone has recorded 132 species of ticks [5]. Ticks are primary parasites of native animals and only about 10% of them act as vectors of domestic animals and human diseases [1]. However, with global warming and climate change, increasing urbanization and environmental disturbances resulting in increased exposure of the human population to native vegetation and zoonotic cycles, this estimate of 10% [1] may be an underestimate and unreliable. Ticks not only pose a high economic burden on livestock globally but also have a great impact on public health with infections such as Lyme borreliosis and other zoonotic tick-borne illnesses [1]. The genus *Rickettsia* has been variously classified into either four orthologous groups: the ancestral group (AG), typhus group (TG), transitional group (TRG), and spotted fever group (SFG) rickettsiae [6]; or into five phylogenetic groups: I belli group (BG), II canadensis group (CG), III typhus group (TG), IV spotted fever group II (SFG II), and V spotted fever group I (SFG I) [7].

Bhutan is a country with rich wildlife and a natural forest coverage of over 60% [8]. About 66% of the country’s population reside in rural settings [9,10] and are involved in farming and livestock-related activities. In 2016, Bhutan’s livestock population largely comprised cattle (303,374), followed by yaks (49,617), goats (39,513), cats (33,866), dogs (28,630), horses (18,890), pigs (15,324) and sheep (11,277) [11]. These populations fluctuate as per the 2023 report, with increasing poultry and pig populations and a reducing bovine population [12]. Most of these domestic animals in Bhutan are free-range or pets (almost exclusively cats and dogs) but are commonly stray by habit. These factors, especially in a rural environment, make animals (both wild and domestic) and human interactions conducive to tick-borne zoonoses. Understanding the diversity and distribution of ticks and their host species is crucial to understand their zoonotic potential locally. There is currently no information regarding the tick fauna and associated rickettsial bacteria in Bhutan. Therefore, this study was undertaken to generate preliminary information on ticks infesting domestic animals in Bhutan and the rickettsial bacteria with which they are associated.

## 2. Materials and Methods

### 2.1. Study Design and Sample Size

This study was caried out as a descriptive study in which ticks were collected from domestic animals residing in eight of the twenty administrative districts of Bhutan. The eight districts selected based on probability proportionate to size sampling, were the study sites of a previous study on seroprevalence of rickettsial diseases in humans and domestic animals [13,14]. A definite sample size was not pre-determined for this study as ticks were collected opportunistically along with animal blood samples (for a previous study) to obtain preliminary data.

### 2.2. Tick Collection, Storage and Transportation

From January to March 2015, ticks (and fleas) were collected by livestock field staff from the domestic animals residing in the study sites (one rural and one urban) of the eight districts. Collected ticks were directly placed into 99.9% ethyl alcohol and transported at room temperature from different sites to the central laboratory in Thimphu, Bhutan. These were shipped to the Australian Rickettsial Reference Laboratory (ARRL) at room temperature for further processing. From the ARRL, ticks were shipped to the United States National Tick Collection (USNTC), Georgia Southern University, for morphological identification. After identification, all ticks were shipped back to the ARRL.

### 2.3. Morphological Identification of Ticks

Ticks were identified using previously published descriptions [15,16,17] as well as comparison with the reference specimens stored in the USNTC. Morphological identification of all stages of the ticks was examined on an Olympus SZX16 stereoscopic microscope (Olympus, Tokyo, Japan, https://www.olympus-global.com/technology/design/product/szx16.html, accessed 23 January 2025) by an expert taxonomist at the USNTC.

### 2.4. Processing of Ticks for Rickettsial DNA Testing

#### 2.4.1. Physical Processing

All ticks and fleas were processed individually. Each large tick was cut through its median axis into two equal halves; one half was returned to the primary vial for storage, and the other half was used for DNA extraction. Tiny ticks and fleas were processed whole. The ticks and fleas were placed in a 1.5 mL microtube containing phosphate-buffered saline (PBS) and grounded thoroughly with a motor-driven tissue grinder (Sysmatec laboratory equipment, https://sysmatec.ch/en/produit/motor-driven-tissue-grinder-g50, accessed 23 January 2025).

#### 2.4.2. DNA Extraction

From the grounded product, about 0.5 mL was taken into a new 1.5 mL microtube for DNA extraction. DNA was extracted using the HiYieldTM DNA Mini Kit, YGB100, Real Genomics, Taipei, Taiwan (http://www.real-biotech.com, accessed 20 January 2025) following manufacturer instructions. Similarly, flea DNA was extracted using the Isolate II Genomic DNA isolation kit from Bioline, Australia (http://www.bioline.com/au/isolate-ii-genomic-dna-kit.html, accessed 21 December 2024). Both these extraction methods are well established and routinely used in the ARRL.

#### 2.4.3. Molecular Testing for Rickettsiae

All extracted DNA were screened for *Rickettsia* and *Coxiella* DNA by the real-time quantitative polymerase chain reaction (qPCR) assays targeting the citrate synthase (gltA), and com1 genes, respectively. These protocols were previously described methods [18] and established as the ARRL protocol for routine diagnosis and research. Being genetically similar, spotted fever group (SFG) and typhus group (TG) rickettsiae were tested targeting the gltA gene primers (CS-F 5′-TCG CAA ATG TTC ACG GTA CTT T-3′, CS-R 5′-TCG TGC ATT TCT TTC CAT TGT G-3′) and probe (CS-Probe 5′-FAM TGC AAT AGC AAG AAC CGT AGG CTG GAT G BHQ1-3′) [19]. For *Coxiella*, the com1 gene primers (com1-F 5′-AAA ACC TCC GCG TTG TCT TCA-3′, com1-R 5′GCT AAT GAT ACT TTG GCA GCG TAT TG-3′) and probe (com1-probe 5′-FAM AGA ACT GCC CAT TTT TGG CGG CCA BHQ1-3′) [20] were used. These primers were designed using primer express from Applied Biosystems, California, USA (https://www.thermofisher.com/au/en/home/brands/applied-biosystems.html, accessed 20 December 2024). Samples with threshold cycle (Ct) values of <35 were deemed positive, those between 35 and 40 equivocal (repeated to determine their status), and those > 40 considered as negative, against the respective target DNA. Positive and negative controls were used with each qPCR run.

All qPCR positive samples were subjected to a mixed primer PCR (with two forward and one reverse primer), modified from a previously described nested PCR for detection of gltA and 17 kDa genes for *Rickettsia* [21] (Table 1). DNA bands were observed under 1.5% gel electrophoresis.

### 2.5. DNA Sequencing and Phylogenetic Analysis

PCR products of the samples positive by conventional PCR were shipped at room temperature to Macrogen Inc, a South Korean biotechnology company (https://dna.macrogen.com/eng/, accessed 10 July 2024) for DNA sequencing. Rickettsiae DNA sequences received from Macrogen Inc were subjected to phylogenetic analysis by using the Neighbour-Joining (NJ) method [22] implemented in MEGA version 7 [23]. DNA sequence coding for gltA and 17 kDa genes were aligned using ClustalW [24], followed by manual refinement to ensure positional homology. Phylogenetic trees were constructed based on pairwise evolutionary distances computed using the Kimura 2-Parameter (K2P) model [25], which accounts for differences in transition and transversion rates. The NJ algorithm was employed to infer the phylogenetic tree topology based on the computed distance matrix. Gaps and missing data were treated using the pairwise deletion option to maximize the use of available data for each comparison. The topological robustness of the tree was assessed by bootstrap analysis with 1000 replicates [26]. All analyses were performed in MEGA version 4 under default settings unless otherwise specified.

The DNA sequences were submitted to GenBank and assigned accession numbers from PV815646 to PV815664 against the gltA genes and from PV815665 to PV815680 against 17 kDa gene, as detailed in Table 2.

## 3. Results

Two hundred ticks were sampled from 155 domestic animals including cattle, dogs, goats, horses, yaks, sheep and cats. More than one tick was collected from 35 (23%) of the animals, mainly cattle, dogs and yaks. All the ticks were identified morphologically but only 188 of them were processed for rickettsial DNA testing, with 12 of them retained for educational display. The 200 ticks belonged to twelve different species, the commonest being *Rhipicephalus microplus* (89) followed by *Rhipicephalus haemaphysaloides* (63), *Haemaphysalis* sp. near *ramachandrai* (14), *Haemaphysalis tibetensis* (11), *Haemaphysalis bispinosa* (10), *Haemaphysalis* sp. (4), *Haemaphysalis* sp. near *davisi* (3), *Rhipicephalus sanguineus* (2), *Haemaphysalis shimoga* (1), *Haemaphysalis hystricis* (1), *Ixodes ovatus* (1), and *Amblyomma testudinarium* (1). The commonest cattle tick was *R. microplus*, the most common dog tick was *R. haemaphysaloides* and all the eleven ticks from yaks were *H. tibetensis*. The details of the animals sampled, ticks collected, and tick species identified from different animals are presented in Table 3.

**Figure 1:**
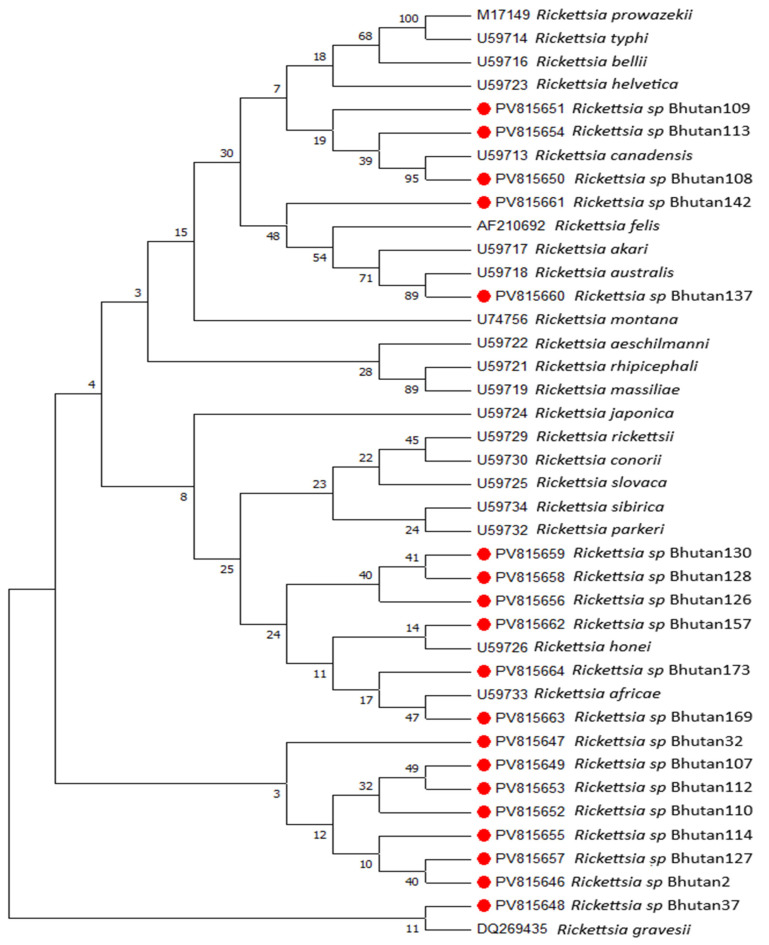
Phylogenetic tree of the rickettsial DNA sequences based on the citrate synthase (gltA) gene. The evolutionary history of the *Rickettsia*. Citrate synthase gene (gltA) identified in Bhutan was inferred using the Neighbour-Joining method [22]. The bootstrap consensus tree inferred from 1000 replicates is taken to represent the evolutionary history of the analyzed taxa [26]. Branches corresponding to partitions reproduced in less than 50% bootstrap replicates are collapsed. The percentage of replicate trees in which the associated taxa clustered together in the bootstrap test (1000 replicates) is shown next to the branches [26]. The evolutionary distances were computed using the Kimura 2-parameter method [25] and are in the units of the number of base substitutions per site. The analysis involved 48 nucleotide sequences. Codon positions included were 1st + 2nd + 3rd + Noncoding. All ambiguous positions were removed for each sequence pair. There was a total of 494 positions in the final dataset. Evolutionary analyses were conducted in MEGA7 [23]. Red dots in the tree indicate the five sequences characterized in this study.

**Figure 2:**
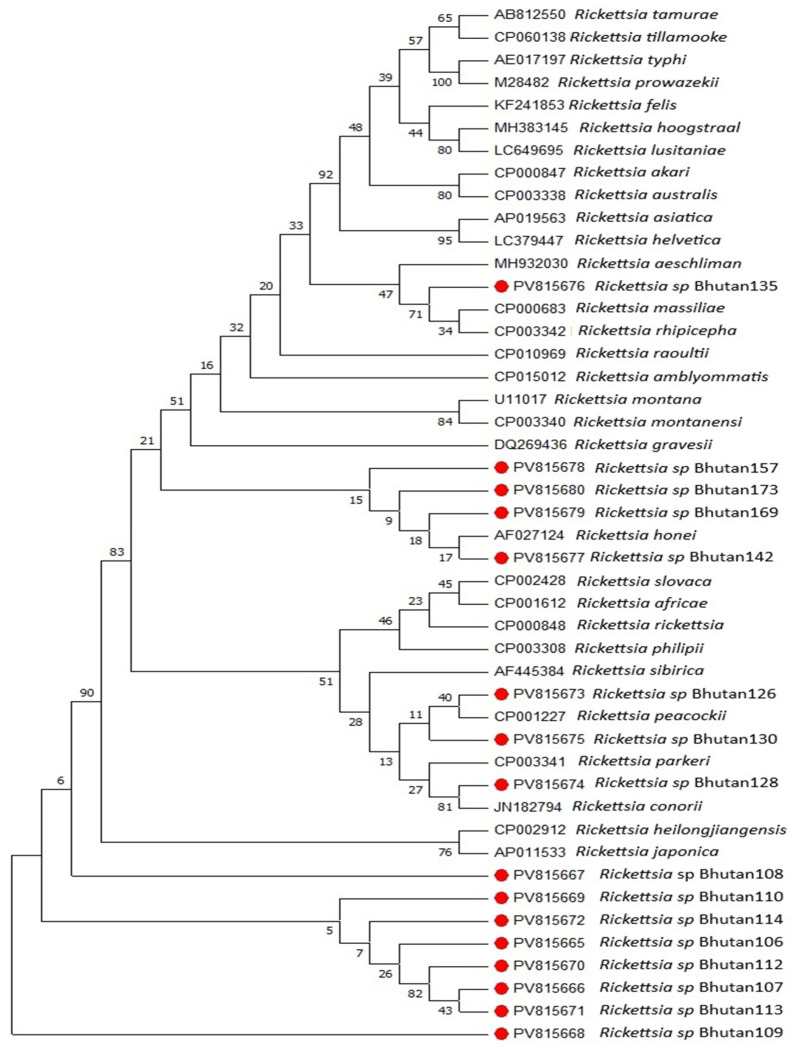
Phylogenetic tree of the rickettsial DNA sequences based on the 17 kDa gene. The evolutionary history of the *Rickettsiae* 17 kDa protein-coding gene (17 kDa gene) identified in Bhutan was inferred using the Neighbour-Joining method [22]. The bootstrap consensus tree inferred from 1000 replicates is taken to represent the evolutionary history of the analyzed taxa [26]. Branches corresponding to partitions reproduced in less than 50% bootstrap replicates are collapsed. The percentage of replicate trees in which the associated taxa clustered together in the bootstrap test (1000 replicates) is shown next to the branches [26]. The evolutionary distances were computed using the Kimura 2-parameter method [25] and are in the units of the number of base substitutions per site. The analysis involved 48 nucleotide sequences. Codon positions included were 1st + 2nd + 3rd + Noncoding. All ambiguous positions were removed for each sequence pair. There was a total of 494 positions in the final dataset. Evolutionary analyses were conducted in MEGA7 [23]. Red dots in the tree indicate the five sequences characterized in this study.

In qPCR, of the 188 ticks, 29 (15%) were positive for *Rickettsia*, and none were positive for *Orientia* or *Coxiella* DNA. In conventional PCR, considering the tick species, twelve (19%) of the *R. haemaphysaloides*, nine (82%) of *H. tibetensis* and eight (9%) of *R. microplus* were positive for rickettsial DNA. DNA sequence analysis of the rickettsiae gltA gene and 17 kDa genes resulted in phylogenetic trees shown in Figure 1 and Figure 2, respectively. Phylogenetic tree analysis showed that the rickettsiae infesting ticks in Bhutanese domestic animals mostly belonged to phylogenetic group V (comprising SFGI) and group II (the canadensis group) based on the recent classification using phylogenetic analysis.

With respect to the gltA gene, four DNA sequences from yak ticks (*H. tibetensis*) and three from cattle ticks (*R. microplus*) formed a different group which aligned with *R. gravesii*, three other sequences from yak ticks (*H. tibetensis*) aligned with *R. canadensis*, those from dog ticks (*R. haemaphysaloides*) aligned with *R. honei*, *R. africae*, *R. felis*, *R. akari*, *R. australis* and *R. japonica*, and the lone sequence from a goat tick (*R. haemaphysaloides*) aligned with *R. africae*. With respect to the 17 kDa gene, all sequences from yak ticks aligned with *R. japonica* and *R. heilongjiangensis*, while those from dog ticks showed variability by aligning with *R. conorii*, *R. peacockii*, *R. honei*, *R. massiliae* and *R. rhipicephali*.

## 4. Discussion

This study presents the first report on Bhutan’s tick fauna in domestic animals and rickettsial bacteria associated with them. Based on these findings, ticks of the genus *Rhipicephalus* and genus *Haemaphysalis* were the dominant ticks parasitizing domestic animals in Bhutan. Ticks from domestic animals contained rickettsiae that belonged mostly to Group V of the phylogenetic classification of rickettsiae, belonging to the SFG I group. However, with no previous studies or references on ticks from Bhutan, it proved to be a daunting task even for tick experts to identify Bhutanese ticks, complicated by the existing disagreements and continuing revisions of tick taxonomy [3].

In India, a country with which Bhutan shares the largest border, the burden and damages caused by ticks and tick-borne diseases (TTBDs) to livestock were reported to be high [27]. Although such burden assessment has never been conducted in Bhutan, it could be significant since it is common to see many domestic animals parasitized by ticks throughout the four seasons. An Indian study identified more than one tick species from the same animal host and disease transmission potential was reported for ticks of *Boophilus*, *Haemaphysalis*, *Hyalomma*, *Rhipicephalus* and *Argas* genera [28]. Similarly, more than one tick species was identified from domestic animals in this study. China, the only other country bordering Bhutan, in the north, has recorded 117 species of ticks of different families, representing 13% of the world’s tick species. The same study recorded several hotspots or belts of provinces with abundant ticks including the Tibetan province that adjoins Bhutan in the north [29]. Yaks are unique to the cold Himalayan mountainous areas of Bhutan bordering the Tibetan province of China. The identification of all ticks from the Bhutanese yaks in this study as *H. tibetensis* was consistent with the report of *H. tibetensis* being found only in the Tibetan province of China [29], although the authors did not specify the host animals. Yaks have a significant, and sometimes exclusive, role in the livelihood of the nomadic population of Bhutan since they depend on yaks for transportation, food (meat, butter, cheese and whey), dung, hide, hair and fibre. The knowledge of ticks and tick-associated diseases and their control would be highly valuable to this population for preserving their livelihoods. Other parts of Bhutan have more diverse sources of income including agriculture and various domestic animals compared to the highlanders. However, public health interventions on ticks and associated diseases could benefit all farmers and related populations in the country.

Elaborate studies on rickettsial species in south Asian countries are limited. Studies from India report *R. japonica*, *R. africae*, *R. sibirica*, *R. rickettsii*, *R. honei*, *R. conorii*, *R. typhi*, *R. raoulti*, *R. parkeri*, and *R. conorii* as some of the rickettsiae species detected in India [30,31]. *R. honei* was also identified as a cause of infection in travellers from Nepal [32]. These rickettsiae species identified in India and Nepal are also similar to those isolated from ticks in southwest China [33]. Due to the small number of samples and few species of ticks identified, no definite comparison with other regional and international published list of ticks and rickettsiae can be made from our study. Additionally, some of the relations in the phylogenetic tree showed low bootstrap values. Low bootstrap values indicate weak support for a branching pattern, meaning those relationships are uncertain and should be interpreted with caution. They do not imply the phylogeny is wrong, but rather that the data lack a sufficient signal to resolve that split confidently. Such nodes are best treated as unresolved, and stronger conclusions may require more data, additional taxa, or complementary methods in future studies. This uncertainty is also shown in the two genes (gltA and 17 kDa) implicating different species from the same sequence in some of the sequences analyzed. However, this data should prompt larger future studies aimed at a definitive list of tick species and their distribution with their associated rickettsiae in Bhutan.

These findings should also contribute to recognizing the medical and veterinary significance of ticks and associated pathogens and initiate public health programmes, diagnostic and research capacities through a collaborative One Health approach. Exploration of other diseases transmitted by ticks such as Lyme disease, anaplasmosis, ehrlichiosis, bartonellosis, babesiosis, and several other emerging infections may be beneficial to prevent morbidity and mortality in the rural population and increased leisure activities, including tourists, in the forests. Future research should focus on hot-spot mapping of the ticks and associated rickettsial (and other tick-associated pathogens) agents according to topography, climate, land use patterns, vegetation and host distribution in different parts of the country. Tick-associated harm to livestock is important and research should be carried out to monitor this since livestock is the main source of livelihood for many of the rural Bhutanese population.

## 5. Conclusions

In this first report of ticks and associated rickettsia in Bhutanese domestic animals, the commonest ticks were of the genus *Rhipicephalus* and *Haemaphysalis*. Rickettsiae isolated from these ticks were similar to those parasitizing domestic animals in the neighbouring regions of the Himalayas and other Asian countries. There may be considerable burden of ticks and rickettsia (and other tick-borne infections) to domestic animals with potential human transmission and Bhutan would benefit from a large-scale study on tick-associated harm assessment in livestock and human public health.

## 6. Limitations of the Study

This study has a few important limitations. First, the sample size was small since it was only undertaken as an opportunistic sampling during a previous study. Second, only morphological identification of the ticks was performed in this study, not molecular identification. Third, the study was performed on ticks collected in 2015, but the findings are the first report for Bhutan and very much valid. Lastly, only two rickettsial genes were sequenced for phylogenetic analysis.

## Figures and Tables

**Table 1:** Primers and probes used in mixed primer PCR for *Rickettsia*.

Target Genes	Primer Sequences
**Citrate synthase (gltA) gene (2 PCRs)**	
Forward 1 (RpCS.780)—479 bp	GACCATGAGCAGAATGCTTCT
Forward 2 (RpCS. 877)	GGGGGCCTGCTCACGGCGG
Reverse RpCS 1258n (both)	ATTGCAAAAAGTACAGTGAACA
**17 kDa gene (2 PCRs)**	
Forward 1—Rr 17k.1p	TTTACAAAATTCTAAAAACCAT
Forward 2—Rr 17k.90p	GCTCTTGCAACTTCTATGTT
Reverse—Rr17k.539n	TCAATTCACAACTTGCCATT

**Table 2:** GenBank accession numbers of each rickettsia isolate from Bhutan.

Scheme	Bhutan Sequence ID	GenBank Seq no.	GenBank Accession no.	Target Gene
1	*Rickettsia* sp. Bhutan2	BankIt2971817 Seq1	PV815646	CS (gltA)
2	*Rickettsia* sp. Bhutan32	BankIt2971817 Seq2	PV815647	CS (gltA)
3	*Rickettsia* sp. Bhutan37	BankIt2971817 Seq3	PV815648	CS (gltA)
4	*Rickettsia* sp. Bhutan107	BankIt2971817 Seq4	PV815649	CS (gltA)
5	*Rickettsia* sp. Bhutan108	BankIt2971817 Seq5	PV815650	CS (gltA)
6	*Rickettsia* sp. Bhutan109	BankIt2971817 Seq6	PV815651	CS (gltA)
7	*Rickettsia* sp. Bhutan110	BankIt2971817 Seq7	PV815652	CS (gltA)
8	*Rickettsia* sp. Bhutan112	BankIt2971817 Seq8	PV815653	CS (gltA)
9	*Rickettsia* sp. Bhutan113	BankIt2971817 Seq9	PV815654	CS (gltA)
10	*Rickettsia* sp. Bhutan114	BankIt2971817 Seq10	PV815655	CS (gltA)
11	*Rickettsia* sp. Bhutan126	BankIt2971817 Seq11	PV815656	CS (gltA)
12	*Rickettsia* sp. Bhutan127	BankIt2971817 Seq12	PV815657	CS (gltA)
13	*Rickettsia* sp. Bhutan128	BankIt2971817 Seq13	PV815658	CS (gltA)
14	*Rickettsia* sp. Bhutan130	BankIt2971817 Seq14	PV815659	CS (gltA)
15	*Rickettsia* sp. Bhutan137	BankIt2971817 Seq15	PV815660	CS (gltA)
16	*Rickettsia* sp. Bhutan142	BankIt2971817 Seq16	PV815661	CS (gltA)
17	*Rickettsia* sp. Bhutan157	BankIt2971817 Seq17	PV815662	CS (gltA)
18	*Rickettsia* sp. Bhutan169	BankIt2971817 Seq18	PV815663	CS (gltA)
19	*Rickettsia* sp. Bhutan173	BankIt2971817 Seq19	PV815664	CS (gltA)
20	*Rickettsia* sp. Bhutan106	BankIt2972213 Seq1	PV815665	17 kDa
21	*Rickettsia* sp. Bhutan107	BankIt2972213 Seq2	PV815666	17 kDa
22	*Rickettsia* sp. Bhutan108	BankIt2972213 Seq3	PV815667	17 kDa
23	*Rickettsia* sp. Bhutan109	BankIt2972213 Seq4	PV815668	17 kDa
24	*Rickettsia* sp. Bhutan110	BankIt2972213 Seq5	PV815669	17 kDa
25	*Rickettsia* sp. Bhutan112	BankIt2972213 Seq6	PV815670	17 kDa
26	*Rickettsia* sp. Bhutan113	BankIt2972213 Seq7	PV815671	17 kDa
27	*Rickettsia* sp. Bhutan114	BankIt2972213 Seq8	PV815672	17 kDa
28	*Rickettsia* sp. Bhutan126	BankIt2972213 Seq9	PV815673	17 kDa
29	*Rickettsia* sp. Bhutan128	BankIt2972213 Seq10	PV815674	17 kDa
30	*Rickettsia* sp. Bhutan130	BankIt2972213 Seq11	PV815675	17 kDa
31	*Rickettsia* sp. Bhutan135	BankIt2972213 Seq12	PV815676	17 kDa
32	*Rickettsia* sp. Bhutan142	BankIt2972213 Seq13	PV815677	17 kDa
33	*Rickettsia* sp. Bhutan157	BankIt2972213 Seq14	PV815678	17 kDa
34	*Rickettsia* sp. Bhutan169	BankIt2972213 Seq15	PV815679	17 kDa
35	*Rickettsia* sp. Bhutan173	BankIt2972213 Seq16	PV815680	17 kDa

**Table 3:** Tick species identified from the seven species of domestic animals in Bhutan.

Animals Sampled (*n* = 155)	No. of Ticks Collected	Tick Species Identified											
		*Rhipicephalus microplus*	*Rhipicephalus haemaphysaloides*	*Haemaphysalis* sp. near *ramachandrai*	*Rhipicephalus sanguineus*	*Haemaphysalis bispinosa*	*Haemaphysalis* sp. near *davisi*	*Haemaphysalis* sp.	*Haemaphysalis shimoga*	*Haemaphysalis hystricis*	*Haemaphysalis tibetensis*	*Ixodes ovatus*	*Amblyomma testudinarium*
Cattle (*n* = 78)	108	79	16	6	0	1	2	2	1	0	0	1	0
Dogs (*n* = 47)	55	7	39	2	2	4	0	1	0	1	0	0	0
Goats (*n* = 10)	11	2	2	0	0	5	0	1	0	0	0	0	0
Horses (*n* = 10)	10	1	2	6	0	0	0	0	0	0	0	0	1
Yaks (*n* = 5)	11	0	0	0	0	0	0	0	0	0	11	0	0
Sheep (*n* = 3)	3	0	2	0	0	0	1	0	0	0	0	0	0
Cats (*n* = 2)	2	0	2	0	0	0	0	0	0	0	0	0	0
Total	200	89	63	14	2	10	3	4	1	1	11	1	1

## Data Availability

The data presented in this study is available on request from the corresponding author.
